# Identification of compendial nonionic detergents for the replacement of Triton X‐100 in bioprocessing

**DOI:** 10.1002/btpr.3235

**Published:** 2022-01-22

**Authors:** Alan K. Hunter, Kamiyar Rezvani, Matthew T. Aspelund, Guoling Xi, Dhanesh Gadre, Thomas Linke, Kang Cai, Sri Hari Raju Mulagapati, Tomasz Witkos

**Affiliations:** ^1^ Department of Purification Process Sciences AstraZeneca Gaithersburg Maryland USA; ^2^ Department of Analytical Sciences AstraZeneca Gaithersburg Maryland USA; ^3^ Department of Analytical Sciences AstraZeneca Cambridge UK

**Keywords:** biotherapeutics, compendial, detergent, Triton ×‐100, virus inactivation

## Abstract

We have systematically investigated six compendial nonionic detergents as potential replacements for Triton ×‐100 in bioprocessing applications. Use of compendial raw materials in cGMP bioprocessing is advantageous for a variety of reasons including material specifications developed to meet stringent pharmaceutical product quality requirements, regulatory familiarity and comfort, and availability from vendors experienced supplying the biopharmaceutical industry. We first examine material properties of the detergents themselves including melting point and viscosity. Process performance and product contact in real‐world bioprocess applications are then investigated. Lastly, we test the detergents in virus inactivation (VI) experiments with recombinant proteins and adeno‐associated virus. Two of the detergents tested, PEG 9 Lauryl Ether and PEG 6 Caprylic/Capric Glycerides, showed favorable properties that make them attractive for use as potential Triton X‐100 replacements. Process performance testing indicated negligible impact of the detergents on product yield, purity, and activity compared to a control with no detergent. Importantly, both PEG 9 Lauryl Ether and PEG 6 Caprylic/Capric Glycerides demonstrated very fast VI kinetics with complete inactivation of XMuLV observed in less than 1 min at a target 1% detergent concentration. Potential advantages and disadvantages of both candidate detergents for use in cGMP bioprocessing are summarized and discussed.

## 1 INTRODUCTION

Owing to their amphiphilic nature, detergents have seen wide use for decades in bioprocessing. Typical applications for detergent use in industrial bioprocessing include virus inactivation,^1^, ^2^, ^3^, ^4^ cell lysis,^5^ endotoxin clearance,^6^ and host cell protein removal.^7^, ^8^

Octyl phenol ethoxylate (Triton X‐100) is among the most commonly employed detergents in the cGMP manufacture of biotherapeutics. Its attractive properties include synthetic origin, well defined composition, low‐hydrophilic lipophilic balance (HLB) while maintaining complete water solubility, low critical micelle concentration (CMC), relatively low viscosity, and the pure material is a liquid at room temperature. Further facilitating its ease of use in bioprocessing, Triton X‐100 is available in compendial grades from high quality raw material vendors experienced supplying the pharma industry. Unfortunately, Triton X‐100 has been identified as an environmental risk due to potential endocrine‐disrupting properties of its degradation products. This has led to its inclusion on the European Chemicals Agency's (ECHA) “candidate list of substances of very high concern (SVHC) for authorization” under the EU REACH regulation framework.^9^ Thus, the industry has been searching for suitable replacements for Triton X‐100.^10^, ^11^, ^12^, ^13^

Compendial monographs are documents maintained by private non‐governmental standard setting bodies such as the United States Pharmacopeia (USP) or the European Pharmacopeia (PhEur). These documents contain chemical and testing information for many pharmaceutical inactive and active ingredients.^14^ There are several advantages to use of compendial raw materials in cGMP bioprocessing. First, the specifications developed and reported in compendial monographs are typically designed to meet stringent pharmaceutical product quality requirements. Second, health authorities frequently participate in monograph development and the wide adoption of these standards results in regulatory familiarity and comfort with use of materials meeting them. Lastly, raw material vendors experienced in supplying compendial raw materials for biopharmaceutical manufacturing understand the strict quality and regulatory requirements of the industry, and expectations associated with a demanding quality audit.

In this work, we report the identification of several detergents with available compendial monographs as possible Triton X‐100 replacements in cGMP bioprocessing applications. We first evaluate physical properties of the candidate detergents, impact on product quality, and process performance for an example protein biotherapeutic. Virus inactivation (VI) studies with protein biotherapeutics are then presented under a range of realistic bioprocess conditions and compared to Triton X‐100 as a control. Lastly, we demonstrate use of one of the detergents for cell lysis and inactivation of an enveloped virus in an adeno‐associated virus (AAV) biotherapeutic process intermediate.

## 2 MATERIALS AND METHODS

### 2.1 Buffer reagents and detergents

Chemicals used for buffer preparation were obtained from Millipore‐Sigma (St. Louis, MO, USA) and JT Baker (Phillipsburg, NJ, USA). Reagent grade Octyl phenol ethoxylate (TX100) was obtained from Amresco (Solon, OH, USA). Multi compendial Polysorbate 80 (PS80) was obtained from JT Baker. PEG 6 Caprylic/Capric Glycerides (G767) was obtained from Croda (Princeton, NJ, USA) meeting an internal Croda testing specification. PhEur and USP/NF grade PEG 9 Lauryl Ether (L9) was obtained from Croda. PEG 25 Propylene Glycol Stearate (MS25) was obtained from Croda meeting an internal Croda testing specification. USP/NF grade PEG 40 Stearate (MS40) was obtained from Croda. PEG 20 Stearyl Ether (S20) was obtained from Croda meeting USP/NF and PhEur compendial monograph specifications. PEG 35 Castor Oil (P35CO) was obtained from Millipore‐Sigma tested to an internal Millipore‐Sigma grade. Note that not all of the detergents used in this work met compendial testing requirements as they were obtained for R&D purposes only. However, with the exception of PEG 25 Propylene Glycol Stearate, all have available PhEur or USP/NF monographs. There may be other compendial monographs available for the detergents tested in this work (e.g., British Pharmacopeia, Japanese Pharmacopeia, etc.) but our search was limited to USP/NF and PhEur.

### 2.2 Model proteins

The two model proteins used in this study were expressed in Chinese hamster ovary (CHO) cells using standard cell culture techniques. A monovalent bispecific human IgG bispecific antibody (BisAb) with an isoelectric point of 9.01 and approximate molecular weight of 150 kDa, and a bispecific Fc‐fusion antibody (BisFusion) with an isoelectric point range of 8.1–9.4 and approximate molecular weight of 280 kDa.

### 2.3 Analytical methods

#### 2.3.1 Protein concentration determination

Protein concentrations of purified samples were determined using a Nanodrop 2000c from Thermo Scientific (Wilmington, DE, USA) with the microvolume pedestal and measurement at a wavelength of 280 nm.

#### 2.3.2 Viscosity measurements

Absolute detergent viscosities as a function of temperature were determined using an mVROC viscometer and associated control software purchased from Rheosense (San Ramon, CA, USA) and integrated Thermo Cube temperature control unit from Solid State Cooling Systems (Wappingers Falls, NY, USA).

#### 2.3.3 Turbidity measurements

Turbidity of diluted detergent and protein solutions were measured using an Orion Aquafast turbidity meter from Thermo Scientific. Solutions were gently mixed end‐over‐end briefly to achieve a homogeneous solution and avoid foaming.

#### 2.3.4 Aggregate determination by size exclusion high performance liquid chromatography

Aggregate levels in purified protein samples were determined by analytical high‐performance size exclusion chromatography (SEC‐HPLC) using a TSKgel G3000SWXL (7.8 mm ID × 30 cm, 5 μm) column obtained from Tosoh Biosciences (King of Prussia, PA, USA) with an Agilent 1260 HPLC system. The HPLC system was operated at 1 ml/min with a mobile phase consisting of 100 mM sodium phosphate, 100 mM sodium sulfate, pH 6.8. Samples (250 μg) were applied to the column neat and the elution profile was monitored at 280 nm using the system spectrophotometer. Aggregate levels were determined as a ratio of peak areas of the early‐eluting aggregate peak(s) and the monomer peak.

#### 2.3.5 Protein bioactivity assay

Protein activity was measured using an in‐house cell based assay. Bioactivity was measured relative to a reference standard before and after the treatment with detergent to assess the impact of detergent treatment on the protein activity.

#### 2.3.6 AAV qPCR viral vector genome titer assay

Samples were treated with DNase I (New England Biolabs, Ipswich, MA, USA) to remove free DNA and with Proteinase K (Invitrogen, Waltham, MA, USA) to break down AAV capsids. Viral DNA was amplified using quantitative polymerase chain reaction (qPCR) with a set of primers and TaqMan probe (5′ 6‐FAM/ 3’ BHQ‐1, Millipore‐Sigma) targeting AAV inverted terminal repeats (ITRs) using the QuantStudio 7 PCR System (Applied Biosystems, Waltham, MA, USA). Purified linearized transgene plasmid was used as a standard to interpolate sample viral genome titers.

#### 2.3.7 AAV infectious titer assay

AAV samples were serially diluted and co‐infected into HelaRC32 cells (ATCC, Manassas, VA, USA) with a human adenovirus 5 standard (ATCC, VR‐1516) in 96‐well plates. After 3 days of incubation at 37°C, DNA was extracted from cells and the virus replication endpoint was detected by qPCR measurement of the replicated viral genome. Median tissue culture infectious dose (TCID50) was calculated by the Spearman‐Kärber method. qPCR was conducted with a set of primers and TaqMan probe (5′ 6‐FAM/ 3’ BHQ‐1, Millipore‐Sigma) targeting AAV inverted terminal repeats (ITRs) using the QuantStudio 7 PCR System (Applied Biosystems).

### 2.4 Product contact and process performance experiments

Samples of harvested cell culture fluid (HCCF) containing BisAb were spiked with detergent and incubated at room temperature (69–75°F) for 24 h. Detergent‐spiked BisAb solution turbidity was measured immediately after detergent spike, and after 1, 2, 3, and 24 h of incubation. After the 24 h detergent incubation, the BisAb solutions were 0.2 micron filtered and purified by lambda light chain affinity chromatography using LambdaFabSelect resin (Cytiva, Uppsala, Sweden) eluted at low pH. Affinity chromatography was performed on an AKTA Avant instrument controlled by Unicorn Software from Cytiva. Product quality of the affinity elution pool was assessed using SEC‐HPLC and protein activity determinations. Chromatography yields were calculated based on A280 measurement of the LambdaFabSelect elution products with respect to the product titer in the HCCF, and accounting for volumetric detergent dilution. BisAb 1 titer in HCCF was determined by an HPLC titer assay using a CaptureSelect Lambda LC column purchased from Thermo Scientific. BisAb 1 concentration in HCCF was quantified by the HPLC elution peak area based on a generated standard curve using purified material.

### 2.5 Virus inactivation experiments

HCCF or neutralized affinity chromatography product containing BisAb or BisFusion were spiked with approximately 8.5 logs of xenotropic murine leukemia virus (XMuLV). Virus inactivation experiments evaluating L9, MS25, MS40, and S20 were conducted using HCCF as the load material, and experiments evaluating G767, PS80, G767 and PS80 combinations, and P35CO were conducted using neutralized affinity chromatography product. Samples of the virus spiked BisAb and BisFusion solutions were collected and used as load and hold controls to determine the rate of virus inactivation without detergent. The load control samples were assayed immediately after initiation of detergent spiking in the virus inactivation samples, while the hold controls were assayed after completion of all reaction time‐points. Detergent stock solutions were made by dissolving detergent into 50 mM Tris pH 7.4. For experiments which included a mixture of detergents, stock solutions were made by dissolving amounts of both detergents into 50 mM Tris, pH 7.4 such that appropriate final concentrations would be reached upon dilution in the protein solution. Protein samples spiked with XMuLV were subsequently spiked to a final intended detergent concentration by dilution with the 1%–10% (w/w) detergent stock solution. Virus inactivation sample solutions were gently mixed end‐over‐end briefly to achieve a homogeneous solution and avoid foaming. Samples were collected at 1, 10, 30, 60, and/or 120 min and diluted 1:50 in McCoy's 5A medium to stop the virus inactivation reaction. Diluted samples and hold controls were assayed for viral concentration using a plaque assay and log reduction values (LRV) were subsequently calculated. The LRVs for MS25 and S20 detergents were calculated based on the load control sample due to an erroneous over‐dilution of the hold control, leading to undetectable virus level in the hold control sample for these virus inactivation experiments. Subsequent virus inactivation experiments using the same BisAb load material and reaction quenching dilution showed minimal virus inactivation in the hold control samples, which suggests an invalid result for the hold control sample in the MS25 and S20 experiments. LRVs for all other detergents shown in this work were calculated based on respective hold controls. Enveloped virus inactivation experiments performed with an AAV product were conducted in a similar manner, with XMuLV spiked into a Capto AVB purified AAV product pool followed by a detergent spike with a 10% (w/w) stock solution to the final detergent concentration. Virus inactivation experiments were performed at an offsite laboratory and therefore the room temperature range stated in Section 2.4 does not apply. As part of the experimental protocol, all initial load temperatures were recorded and found to be in a range of 18–23°C. Due to the small volumes used for the experiments, no active temperature control was employed.

### 2.6 AAV generation and purification

An AAV6 cell paste slurry was generated using standard cell culture techniques and stored at −80°C. Capto AVB resin was sourced from Cytiva. C0SP filters were obtained from Millipore‐Sigma. Sartopore 2 XLG, 0.8/0.2um filters were obtained from Sartorius Stedim Biotech GmbH (Gottingen, Germany).

For detergent lysis, the AAV6 cell paste was diluted to a 10% (w/w) slurry with 20 mM Tris, 200 mM NaCl, 2 mM MgCl2, pH 7.5. A 10% (w/w) detergent stock solution was spiked into cell slurry to bring the final detergent concentration to 0.5% (v/w). Benzonase was spiked to 10 U/ml into cell slurry mixture and mixed slowly at room temperature for 1 h.

AAV purification was performed using a Capto AVB affinity column. Purification was controlled by Unicorn 7.0 software on an AKTA Avant purchased from Cytiva. A C0SP POD filter was flushed with 20 mM Tris, 500 mM NaCl, 2 mM MgCl2, pH 7.5. 10% AAV cell lysate was clarified with C0SP followed by Sartopore 2 XLG, 0.8/0.2um filter. The Capto AVB column was equilibrated with 20 mM Tris, 200 mM NaCl, 2 mM MgCl2, pH 7.5 for 3 column volumes. Clarified cell lysate was loaded with residence time of 6 min. The column was re‐equilibrated with 20 mM Tris, 200 mM NaCl, 2 mM MgCl2, pH 7.5 for 4 CV. Product was eluted from column with 50 mM citric acid, 500 mM NaCl, 2 mM MgCl2, pH 2.5. Eluate pH was adjusted to pH 8.0 with 1 M Tris/HCl, pH 8.0.

## 3 RESULTS AND DISCUSSION

### 3.1 Detergent selection and material properties

Table 1 shows detergents evaluated as part of this work to identify a Triton X‐100 replacement. With the exception of PEG 25 Propylene Glycol Stearate, which has FDA IID status only, all candidates have available USP/NF and/or PhEur compendial monographs. Detergents were selected to span a range of properties including viscosity, melting point, and HLB. Moreover, several of the candidate detergents are approved for use in parenteral drugs, either as an excipient or as an API. This is favorable for use in parenteral drug manufacturing as a robust package of safety testing data is typically associated with such compounds.

**TABLE 1:** Description of detergent properties

Chemical name	Trade or common names	Abbreviation in this work	Compendial monographs and/or FDA IID status^a^	Form at room temperature	Approved parenteral drug use	Hydrophilic‐lipophilic balance^b^
Octyl phenol ethoxylate	Triton X‐100, Octoxynol‐9	TX100	USP/NF, PhEur, FDA IID	Clear colorless liquid	Yes (excipient)^c^	13.5
Polysorbate 80	Tween 80	PS80	USP/NF, PhEur, FDA IID	Clear yellow viscous liquid	Yes (excipient)^d^	15.0
PEG 9 Lauryl Ether	Laureth 9, Polidocanol	L9	USP/NF, PhEur	White semi‐solid cream	Yes (API for vascular disorders)^e^	14.3
PEG 35 Castor Oil	Kolliphor EL	P35CO	USP/NF, PhEur, FDA IID	Clear yellow viscous liquid	Yes (excipient)^d^	12.7
PEG 6 Caprylic/Capric Glycerides	Glycerox 767, Softigen 767	G767	USP/NF, PhEur	Pale yellow liquid	None identified	13.2
PEG 20 Stearyl Ether	Steareth 20	S20	USP/NF, PhEur	White waxy solid	None identified	15.3
PEG 25 Propylene Glycol Stearate	Myrj S25/1	MS25	FDA IID	White semi‐solid cream	None identified	16.0
PEG 40 Stearate	Myrj S40	MS40	USP/NF, PhEur, FDA IID	White waxy solid	None identified	16.7

^a^ Indicates availability of compendial monographs, see Section 2.1 for material grades used in this work. FDA IID is the US Food and Drug Administration Inactive Ingredient Database.

^b^ Information obtained from manufacturer's literature.

^c^ Data from Centers for Disease Control and Prevention.^15^

^d^ Data from FDA Center for Drug Evaluation and Research.^16^

^e^ Data from Merz North America.^17^

**FIGURE 1:**
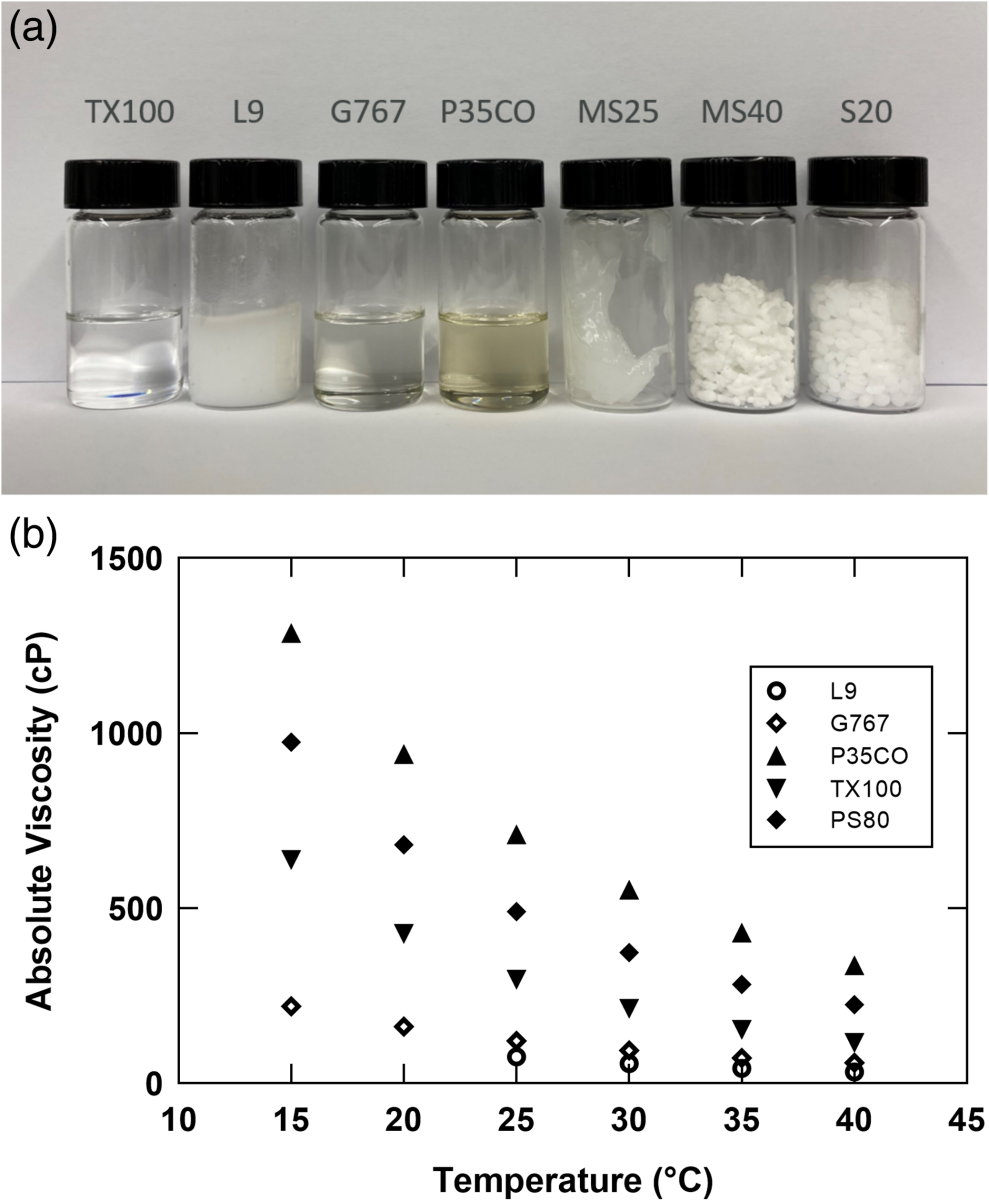
(a) Representative appearance of pure detergents at room temperature (69–75°F) and (b) viscosity of pure detergents as a function of temperature

Figure 1a shows representative images of pure detergent preparations at room temperature in our laboratory, which is maintained between 69 and 75°F. In addition to Triton X‐100, two detergents, G767 and P35CO, are liquid at room temperature. The remainder have a waxy solid or semi‐solid cream like consistency. L9 has a melting point just above room temperature and the pure material was often observed as two phases in our lab under ambient conditions, with a liquid layer on top of a more dense solid layer. Figure 1b shows viscosities of pure detergent preparations as a function of temperature. Interestingly, L9 above its melting point showed the lowest viscosity. G767 also showed a favorable low viscosity profile. While P35CO exhibited the highest viscosity, handling and transfer of this detergent was qualitatively similar to PS80.

**FIGURE 2:**
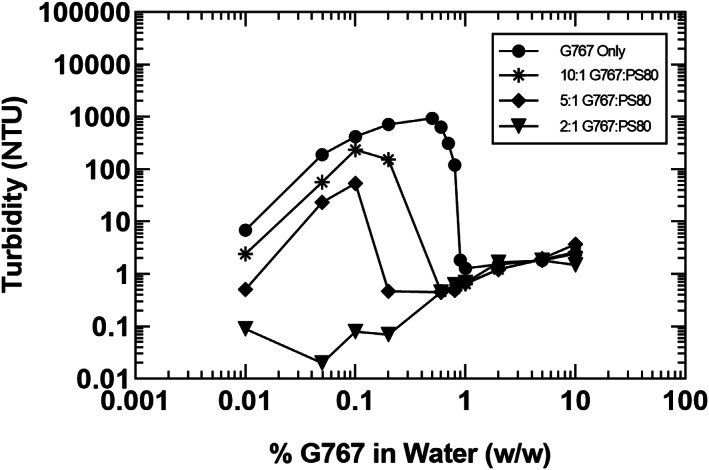
Turbidity of G767 and PS80 solutions as a function of concentration in water at room temperature (69–75°F)

Lastly, it was observed that one of the detergents, G767, showed a marked increase in turbidity when concentrations dropped below 1% (w/w) in water. Figure 2, which shows percent detergent in water versus turbidity at room temperature, illustrates this behavior. As is also shown in Figure 2, it was possible to shift the concentration onset of turbidity to lower concentration by the addition of PS80 in a ratio of 10:1 G767:PS80 avoiding a “cliff effect” where solutions may become turbid near the 1% (w/w) threshold. Increasing ratios of PS80 in G767 solution lowered the concentration at which turbidity was no longer observed, and a 2:1 G767:PS80 ratio completely inhibited turbidity formation in the concentration range evaluated. Preparations of 10% and 1% (w/w) L9 and 10% (w/w) G767 in deionized water were also examined at low‐temperature conditions in a 2–8°C refrigerator and remained completely homogeneous without any observable phase separation over a period of approximately 1 month.

### 3.2 Product contact and process performance

Prolonged detergent contact in HCCF containing BisAb was assessed to understand the potential impact to a realistic biopharmaceutical manufacturing process. Detergent addition to HCCF is frequently employed for virus inactivation and in the authors' experience serves as a worst case scenario for turbidity increase, subsequent purification process performance, and product quality. Figure S1 provided in the supplemental materials displays the turbidity profiles of the different detergent‐incubated samples over a period of 24 h. Notably, MS25 and MS40 detergents at 2% concentration produced substantial increases in solution turbidity after 1 h of incubation, compared to the control condition which was not spiked with detergent. G767 at 1% concentration also produced distinct increase in turbidity, while a 2% concentration did not form turbidity during the 24‐hour incubation. The turbidity formation observed at lower concentrations of G767 in HCCF followed the behavior outlined in Figure 2, and addition of PS80 similarly prevented the turbidity formation in HCCF containing BisAb. TX100, L9, S20, and P35CO detergents were not found to increase HCCF solution turbidity over a 24 h hold at room temperature.

As displayed in Table 2, prolonged incubation in HCCF did not impact affinity chromatography process performance or BisAb product quality relative to a no detergent control for all detergents evaluated. Affinity chromatography product yields were consistent with control conditions, which were not spiked with detergents, and product quality by SEC‐HPLC showed consistent monomer purity. The consistent chromatographic behavior we observed may be expected due to lack of significant non‐specific binding of non‐ionic detergents to typical agarose‐based mAb affinity media, but impact to performance needs to be further evaluated for other separation media. While the impact of detergent on 0.2 μm filtration throughput and product recovery was not specifically evaluated in this study, the load materials were passed through 0.2 μm polyethersulfone membranes prior to affinity chromatography process performance and product quality studies referenced in Table 2. No obvious impact to filtration or process performance was observed. Additionally, no impact on BisAb bioactivity was observed after prolonged contact with detergents. However, further consideration for MS40 was abandoned due to the relatively high increase in turbidity observed during product contact.

**TABLE 2:** BisAb product quality and affinity chromatography performance after prolonged detergent contact in HCCF

Experiment 1^a^	Yield (% by A280)	Monomer (% by SEC)	Aggregate (% by SEC)	Fragment (% by SEC)	Bioactivity (% relative potency)
Control affinity‐capture product^b^	83	95.2	3.5	1.2	96
2% TX100	86	95.8	3.1	1.1	100
2% L9	86	96.1	2.9	1.0	97
2% S20	86	96.1	2.9	1.0	92
2% MS25	86	95.6	3.3	1.1	95
2% MS40	85	95.7	3.2	1.2	93

Experiment 2^a^	Yield (% by A280)	Monomer (% by SEC)	Aggregate (% by SEC)	Fragment (% by SEC)	Bioactivity (% relative potency)
Control affinity‐capture product^b^	93	96.3	2.2	1.5	85
1% G767	92	96.6	2.2	1.2	94
1.5% G767	92	96.3	2.4	1.3	96
2% G767	92	96.2	2.3	1.5	96
1% G767, 0.5% PS80	92	96.4	2.4	1.2	87
2% P35CO	90	96.1	2.5	1.4	—^c^

^a^ Two separate sets of experiments were performed with two distinct lots of BisAb HCCF material.

^b^ HCCF load material without detergent incubation held at same conditions.

^c^ Not tested.

### 3.3 Virus inactivation studies

**FIGURE 3:**
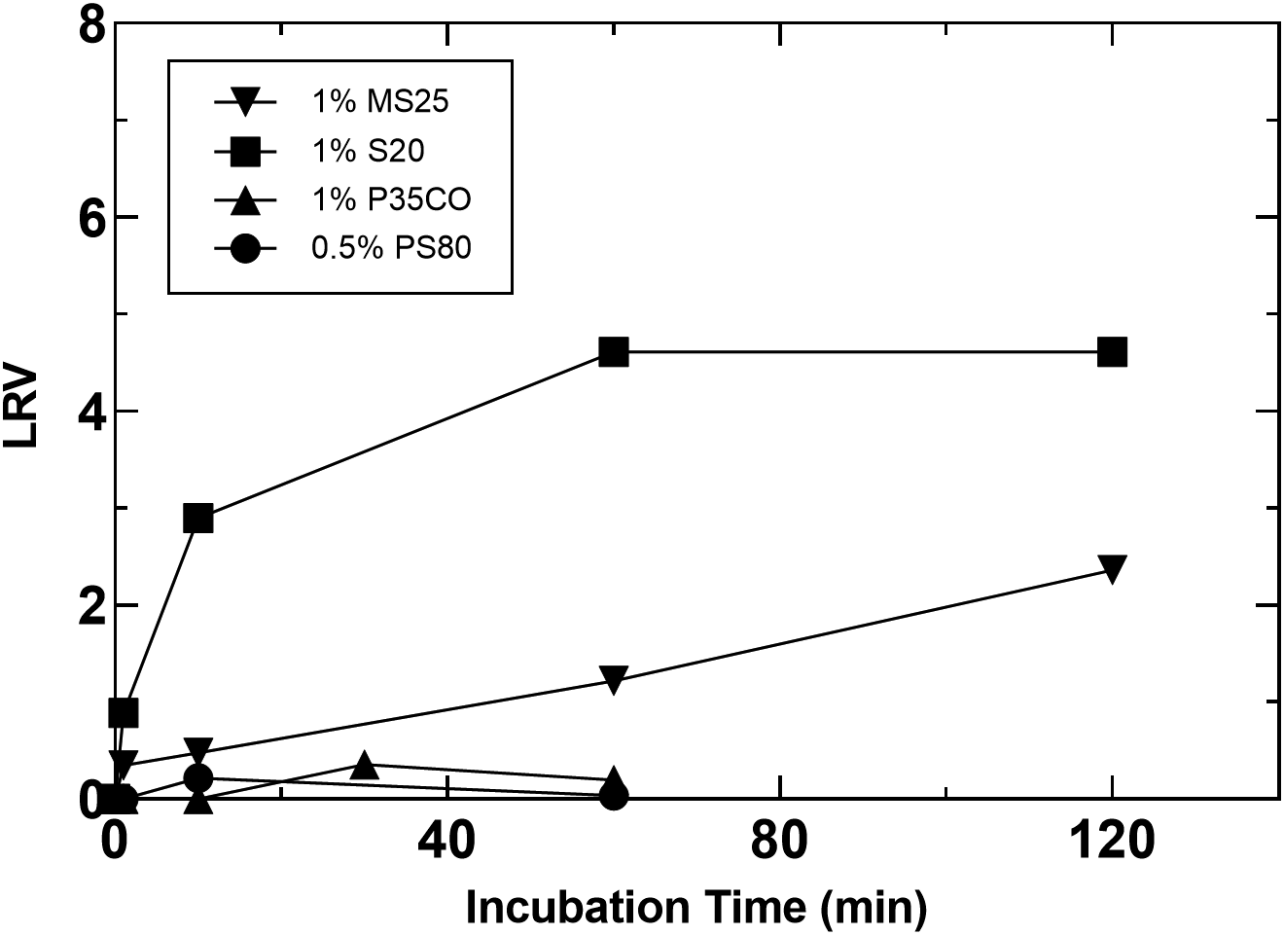
XMuLV virus inactivation kinetics for MS25, S20, P35CO, and PS80. MS25 and S20 experiments were performed in BisAb HCCF at an initial recorded temperature of 23°C. P35CO and PS80 experiments were performed in BisAb affinity capture product at an initial recorded temperature of 18°C

**FIGURE 4:**
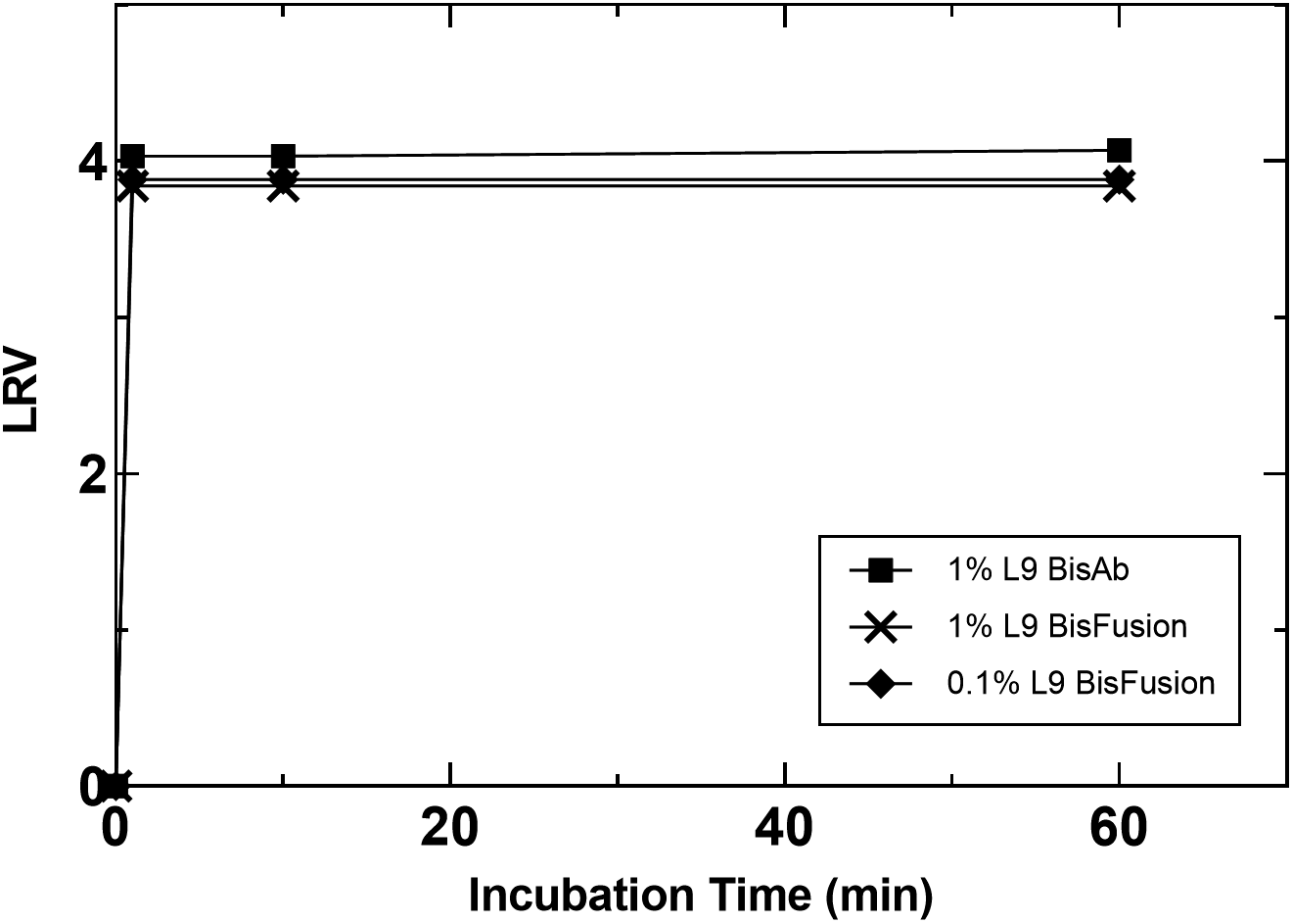
XMuLV virus inactivation kinetics for L9 with two model proteins. Virus inactivation reactions were conducted in HCCF at an initial recorded temperature of 22°C

A summary of the virus inactivation studies performed with the BisAb and XMuLV as a model enveloped virus is provided in Table 3. Figure 3 shows results of VI kinetics for detergents for which slow or no inactivation was observed. These were MS25, S20, and P35CO, which were not considered further, and PS80, which was evaluated to confirm its lack of significant virus inactivation capability alone. In contrast to this, two detergents showed exceptionally fast inactivation kinetics, these were L9 and G767. Figure 4 shows inactivation curves for L9 and displays the capability of L9 to provide fast inactivation in two distinct protein load materials. L9 achieved complete virus inactivation to below the limit of detection (LOD), represented as greater than 4.0 LRV in BisAb HCCF, and greater than 3.8 LRV in BisFusion HCCF, within 1 min at 1% concentration. It was also tested with the BisFusion protein at 0.1% and similarly achieved virus inactivation to below the LOD within 1 min. These results clearly demonstrate L9 robustly achieves virus inactivation with very fast kinetics. As such, it has high potential to replace Triton X‐100 for this bioprocessing application.

**TABLE 3:** Summary of detergent virus inactivation performance

Surfactant condition	Viral inactivation kinetics	Load material used for VI experiment	Recorded temperature of load at start of experiment (°C)	Data reference
1% TX100	1 min^a^	BisAb HCCF	22	Data not shown
0.5% PS80	None^b^	BisAb affinity capture product	18	Figure 3 (●)
1% L9	1 min^a^	BisAb HCCF	22	Figure 4 (∎)
1% P35CO	None^b^	BisAb affinity capture product	18	Figure 3 (▴)
1% G767	1 min^a^	BisAb affinity capture product	18	Figure 5 (▴)
1% G767 + 0.5% PS80	60 min^c^	BisAb affinity capture product	18	Figure 5 (○)
1% G767 + 0.1% PS80	1 min^a^	BisAb affinity capture product	18	Figure 5 (◆)
1% S20	60 min^c^	BisAb HCCF	23	Figure 3 (∎)
1% MS25	Slow^d^	BisAb HCCF	23	Figure 3 (▼)

^a^ Complete virus inactivation within 1 min after virus spike.

^b^ No significant decrease in virus level compared to controls over entire experiment time‐course.

^c^ Complete virus inactivation in between 10 and 60 min time‐points.

^d^ Incomplete virus inactivation within 60 min time‐course.

**FIGURE 5:**
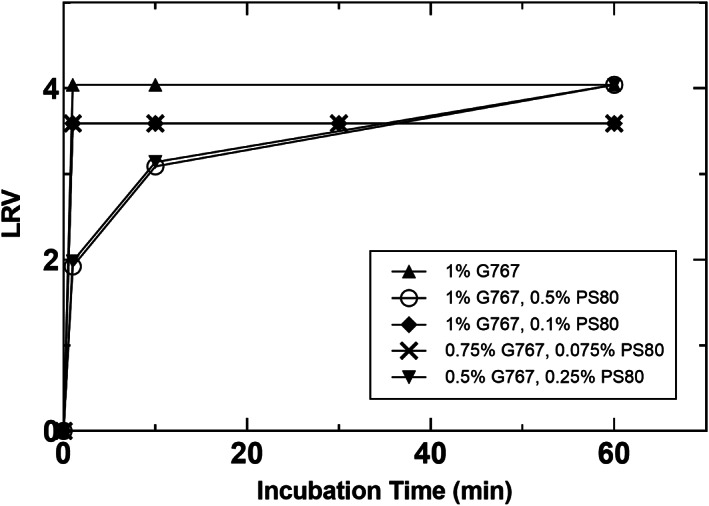
XMuLV virus inactivation kinetics for G767 with varying amounts of PS80. Virus inactivation reactions were conducted in BisAb affinity capture product at an initial recorded temperature of 18°C

Figure 5 shows VI studies performed with G767, or G767 and PS80. As is shown in Figure 5, G767 at 1% concentration with no PS80 showed very rapid inactivation kinetics, achieving greater than 4.0 LRV within 1 min. As is also shown in Figure 5, when 0.5% PS80 is added in addition to 1% G767, VI kinetics slow considerably, achieving complete inactivation to below the LOD only after 60 min. This result was unexpected and suggests PS80 is actually protecting the virus in some fashion given G767 was maintained constant at 1% concentration. The exact mechanism by which PS80 may protect the virus is not known and somewhat counterintuitive given it does possess alkyl chains which presumably may disrupt the virus envelop over time. The impact of PS80 was reproducible, showing nearly identical trends when maintained at a 2:1 ratio G767:PS80 in two different experiments as is also reflected in Figure 5. Furthermore, decreasing the amount of PS80 to a 10:1 G767:PS80 ratio restored the fast virus inactivation kinetics, to below the LOD within 1 min, for 1% and 0.75% G767 conditions, as illustrated in Figure 5. Of note, virus inactivation experiments evaluating G767 and PS80 individually and in combination were performed in neutralized affinity chromatography product rather than HCCF due to recent literature outlining virus inactivation capability of PS80 when spiked into CHO cell culture harvest, due to generation of PS80 degradation products.^12^ Lastly, it should be noted that differences in the max LRV observed for different experiments shown in Figures 4 and 5 simply reflect experiments with separate hold control samples, where minor differences in measured virus concentration will ultimately be reflected as a change in the maximum LRV that can be quantified.

### 3.4 AAV experiments

Table 4 shows results of L9 cell lysis studies for the recovery of AAV6. TX100 was included as a control in these studies. As can be seen from the table, comparable AAV titer values were obtained in the crude lysate for both L9 and TX100, indicating L9 is an effective detergent for cell lysis. To examine whether AAV was active after exposure to L9, material from detergent lysis studies was purified using Capto AVB affinity chromatography and both titer and AAV infectivity were measured in the affinity purified pool. Again, comparable AAV titer and infectivity results were obtained regardless of lysis method used, indicating AAV is stable in the presence of L9.

**TABLE 4:** Detergent lysis efficiency and impact on AAV titer

Cell lysis method	Lysate AAV Titer by qPCR (log_10_ vg/ml)	CaptoAVB Eluate AAV Titer by qPCR (log_10_ vg/ml)	Capto AVB Eluate AAV Titer by Infectivity (log10 TCID_50_/ml)
0.5% TX100	11.03	12.2	—^a^
0.5% L9	11.04	12.2	10.5
0.5% TX100^b^	11.05	11.8	9.8

^a^ Not tested.

^b^ Experiment performed with a different lot of cell paste.

**FIGURE 6:**
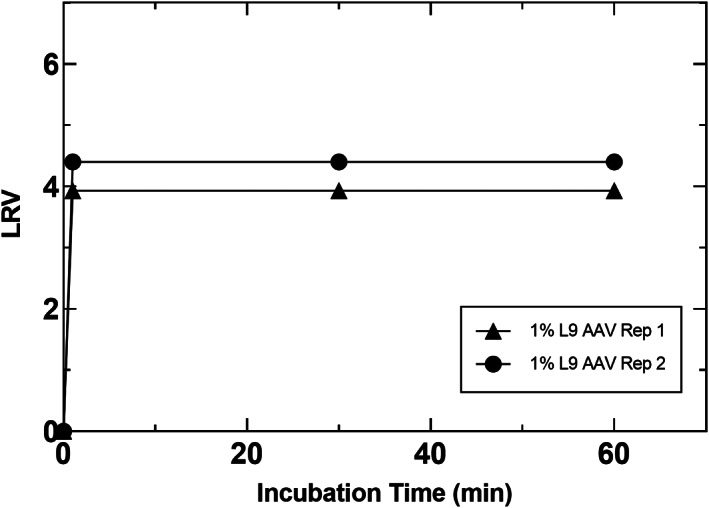
Inactivation of XMuLV in an AAV product pool at an initial recorded temperature of 20°C

Virus inactivation using L9 detergent was performed in AAV at 1% detergent concentration as shown in Figure 6. As can be seen in the figure and similar to prior observations with recombinant proteins, complete inactivation to below the LOD is achieved within 1 min. Inactivation was also tested at 0.1% detergent concentration with AAV and the results are shown in supplemental material Figure S2. At this concentration 1 of the 2 replicates achieved complete inactivation between 1 and 30 min. Nonetheless, the results indicate L9 is very robust from a VI perspective at the target concentration of 1%, and exhibits fast kinetics even at 1/10 the target concentration. Data provided in Table S1 in the supplemental materials also shows that AAV titer and infectivity is comparable when purified material is spiked with TX100 or L9, and subjected to a similar incubation procedure as the virus inactivation experiments. Taken together, the data presented here with AAV demonstrates L9 can inactivate adventitious or endogenous enveloped viruses in a non‐enveloped viral therapeutic or vaccine.

## 4 SUSTAINABILITY CONSIDERATIONS

Neither L9 nor G767 are listed as SVHCs by ECHA under the EU's REACH regulations. However, the use and disposal of any chemical in a manufacturing process is governed by local regulations and ordinances, where required practices can vary greatly by country and region. Therefore, prior to use of either L9 or G767 in a manufacturing setting, an evaluation should be performed to ensure compliance with all such regulations.

## 5 CONCLUSIONS AND RECOMMENDATIONS

We have systematically investigated six compendial detergents as potential replacements for Triton X‐100 in bioprocessing applications. Two of them, L9 and G767, were identified as having favorable properties and good potential for this purpose. Both showed very fast virus inactivation kinetics, and L9 was also shown to be suitable for cell lysis in an AAV application. The other four detergents investigated in this study were excluded from further consideration due to slow VI kinetics as this was a critical requirement for our applications.

Both L9 and G767 have advantages and disadvantages, which are summarized in Table 5. Overall, we would give a minor advantage to L9, provided the necessary equipment is available either to gently warm the pure detergent slightly above room temperature to facilitate handling in a liquid state, or thoroughly mix and pump the viscous semi‐solid consistency observed at room temperature. While we were unable to obtain a viscosity measurement for L9 below 25°C using the Rheosense instrument, suggesting the viscosity is above 100,000 cP, in our lab it was nonetheless possible to effectively transfer it below this temperature using a conventional peristaltic pump (see supplemental Video S1 shot at 22°C for additional details). For room temperature handling of pure L9, we would recommend a heavy duty mixer with a high torque motor and high viscosity impeller such as an anchor or VISCO JET be used to first homogenize the material as it appears to separate into regions of low and high density on standing. To pump L9 at room temperature following thorough mixing, a heavy‐duty positive displacement pump suitable for very high viscosities such as a peristaltic or progressive cavity pump is recommended.

**TABLE 5:** Advantages and disadvantages of L9 and G767 as Triton X‐100 replacements in bioprocessing

	Advantages	Disadvantages
L9	Approved for parenteral use No turbidity issues Fast VI kinetics Compendial monographs available Multiple suppliers Relatively low price Low 280 nm absorbance	Unfavorable material handling of pure detergent: semi‐solid cream consistency at room temp (can be transferred at room temperature but requires thorough mixing and pump suitable for extremely high viscosities, see supplemental Video S1 for additional details)
G767	Favorable material handling: liquid at room temperature, lower viscosity Fast VI kinetics Compendial monographs available Multiple suppliers Relatively low price Low 280 nm absorbance	Turbid at low concentrations PS80 combination adds complexity No documented parenteral use

## AUTHOR CONTRIBUTIONS

**Alan Hunter:** Conceptualization (lead); formal analysis (equal); investigation (equal); methodology (equal); supervision (equal); writing – original draft (equal); writing – review & editing (equal). **Kamiyar Rezvani:** Data curation (equal); formal analysis (equal); investigation (equal); methodology (equal); visualization (equal); writing – original draft (equal); writing – review and editing (equal). **Matthew Aspelund:** Conceptualization (supporting); formal analysis (equal); investigation (equal); methodology (equal); supervision (equal); writing – original draft (equal); writing – review and editing (equal). **Guoling Xi:** Data curation (supporting); investigation (supporting); writing – original draft (supporting). **Dhanesh Gadre:** Data curation (supporting); investigation (supporting); methodology (supporting); writing – original draft (supporting). **Thomas Linke:** Investigation (supporting); methodology (supporting); writing – original draft (supporting). **Kang Cai:** Data curation (supporting); formal analysis (supporting); investigation (supporting); methodology (supporting); writing – original draft (supporting); writing – review and editing (supporting). **Sri Hari Raju Mulagapati:** Methodology (supporting); writing – original draft (supporting). **Tomasz Witkos:** Methodology (supporting); writing – original draft (supporting).

## Supporting information


**Figure S1** HCCF solution turbidity with prolonged detergent contact at room temperature
**Figure S2**. Inactivation of XMuLV with 0.1% L9 in an AAV product pool

**Table S1** Purified AAV stability and infectivity after 60‐minute incubation with detergent

**Video S1** Demonstration of pure L9 transfer at 22°C using a peristaltic pump

## Data Availability

The data that support the findings of this study are available from the corresponding author upon reasonable request.
