# Supplementary material for: Identification of compendial nonionic detergents for the replacement of Triton X‐100 in bioprocessing
Source: Biotechnol Prog. 2022 Jan 22;38(2):e3235. doi: 10.1002/btpr.3235 (PMC9285696; doi:10.1002/btpr.3235)
Supplement: Supplementary file 1 — Figure S1 HCCF solution turbidity with prolonged detergent contact at room temperature Figure S2. Inactivation of XMuLV with 0.1% L9 in an AAV product pool [file BTPR-38-0-s001.pdf]

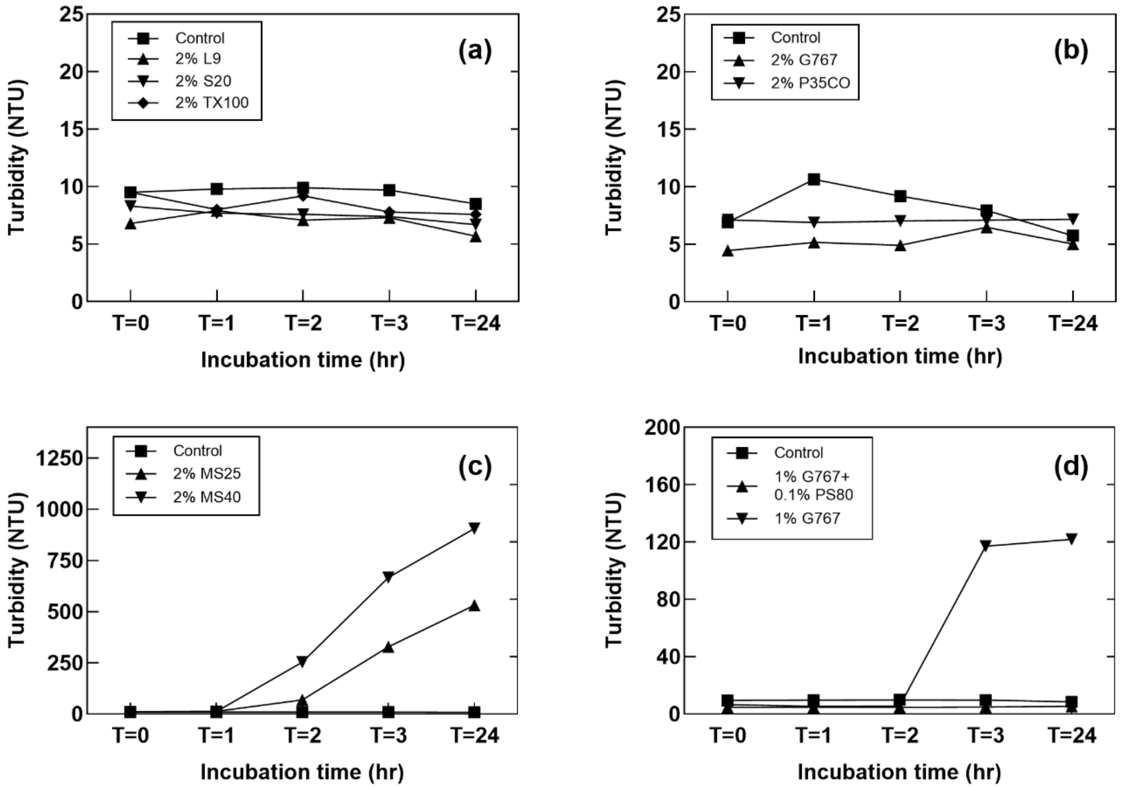

Figure S1. HCCF solution turbidity with prolonged detergent contact at room temperature

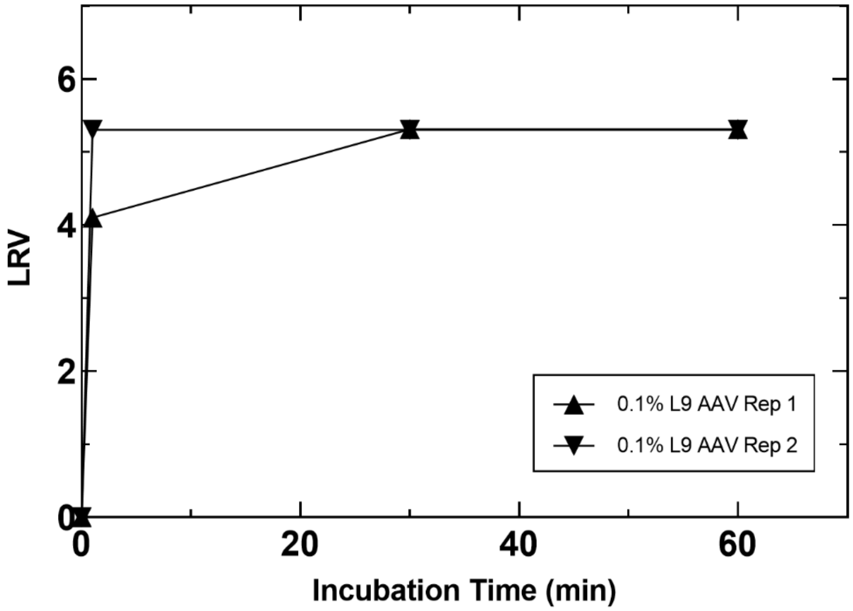

Figure S2. Inactivation of XMuLV with 0.1% L9 in an AAV product pool
