# Supplementary material for: Identification of compendial nonionic detergents for the replacement of Triton X‐100 in bioprocessing
Source: Biotechnol Prog. 2022 Jan 22;38(2):e3235. doi: 10.1002/btpr.3235 (PMC9285696; doi:10.1002/btpr.3235)
Supplement: Supplementary file 2 — Table S1 Purified AAV stability and infectivity after 60‐minute incubation with detergent [file BTPR-38-0-s003.pdf]

Table S1

Purified AAV stability and infectivity after 60-minute incubation with detergent

Detergent Incubation Condition[a]	AAV Titer by qPCR (log_{10} vg/mL)	AAV Titer by Infectivity (log_{10} $TCID_{50}$/mL)
0.1% L9	11.2	9.13
1.0 % L9	11.1	9.30
0.1% TX100	11.1	9.63
1.0% TX100	11.2	9.63

[a] Capto AVB purified AAV samples spiked with detergent, incubated at room temperature, and diluted 1/50 in 50mM Tris, 200mM NaCl, 2 mM MgCl, pH 7.5 solution after 60 minutes
